# Surgical Clinic at Rush Medical College

**Published:** 1893-04

**Authors:** John B. Hamilton

**Affiliations:** Professor of the Principles of Surgery and Clinical Surgery, etc.; Chicago


ATLANTA
MEDICAL AND SURGICAL JOURNAL.

VOL. X. APRIL, 1893. No. 2.

EDITED BY
LUTHER B. GRANDY, M. D., AND MILLER B. HUTCHINS, M. D.,
WITH THE CO-OPERATION OF
H. V. M. MILLER, M. D., LL. D., VIRGIL O. HARDON, M. D.,
FLOYD W. McRAE, M. D., AND ARTHUR G. HOBBS, M. D.

### *CLINICAL LECTURE.*

### SURGICAL CLINIC AT RUSH MEDICAL COLLEGE.

By JOHN B. HAMILTON, M. D.,
Professor of the Principles of Surgery and Clinical Surgery, etc.
CHICAGO.

### SUBACUTE SYNOVITIS.

GENTLEMEN—This patient's leg and knee have been paining him for eight days. There is no history of an injury. There is very little difference in the size of the two knees. The patient says about a week ago the knee commenced to pain him, since which time he has been unable to go up or down stairs or walk without pain. There is a history of the other knee having been similarly affected a short time ago. There is no effusion in the synovial sac, although the symptoms would seem to indicate that it was a mild inflammation of the synovial membrane, therefore a subacute synovitis.

For the relief of the inflammation at this stage it will be well to paint the knee with tincture of iodine and apply a bandage in order to fix the motion of the joint as much as possible. Immovable dressings are an objection to cases of this kind at this time, inasmuch as they tend to produce false ankylosis. Massage or friction of the joint answers a much better purpose than fixing the joint positively by a plaster of paris or silicate dressing. The use of iodine externally is to diminish the hyperemia in the joint, and wherever any infection may be present it is a strong anti-bacillary agent.

### FALSE ANKYLOSIS.

This little girl hurt her elbow nine weeks ago by falling. We find still a discoloration about the elbow joint, and it seems a little wider between the condyles than normally. The ulna and radius are in their correct positions, but I find an inability to extend the arm, and on making an effort to do so I find it is opposed by the biceps and the other muscles that serve to flex the arm. It is impossible to extend the arm on account of the action of the flexor muscle. We have to deal with a case of false ankylosis. It is to be distinguished from true or bony ankylosis by the fact that the motion between the joint or synovial surface remains normal. For instance, I can rotate the bone by grasping the wrist. By rotation I feel the head of the radius moving in its socket. I find also that the ulna moves correctly and the obstruction to extension is muscular. We have contracture of the tendons, doubtless due to an injury. This condition of the tendons is one that we sometimes encounter as a complication after fractures. It is a serious complication, because ankylosis remains quite as permanent and enduring as if it were true ankylosis whereby the bony tissues united the joint.

The only treatment for this case, and all such cases, consists in early passive motion under an anæsthetic, if necessary, in order that full motion of the joint may be restored. As it has only been nine weeks since the date of the original injury there remains but little doubt that we can under an anæsthetic extend the arm fully and restore the motions of the joint. Having once forcibly broken up the adhesions—for the tendons are bound down to the inter-muscular septa by adhesions due to the primary violence which has been received—the patient will have free motion of the arm. The child should, therefore, be anæsthetized and the arm extended.

When a tendon is temporarily shortened by reason of contraction, we term it contracted tendon. If that shortening is permanent we term it a contractured tendon, an arbitrary application being thus given to those terms.

We treat contractured tendons by breaking up the adhesions or by tenotomy (or cutting of the tendon) according to the nature of the case, the length of time the contraction has existed, and the condition of the tendon itself at the time of the operation. I think the cases that give one more trouble than any other are those of fracture of the forearm where the flexor and extensor tendons have their sheaths bound down by adhesions, owing perhaps to an injury received at the time the bones were fractured. These cases require constant care. The fingers must be every day flexed and extended, otherwise there will be a permanent flexion of them, because the flexor tendons are the stronger to overcome, and the fingers will gradually become closed, as you see in the hand of this patient. The tendons can only be moved by section if they have been permanently contractured. Therefore all fractures of the lower part of the radius require that passive motion should be early instituted in order that the tendons may not become adherent. This extension should be made gradually by tiring the muscle out, as it were, then you will find that the arm can be extended. The patient is now anæsthetized, and I will now flex the arm as far as possible, pronate and supinate the hand, put it through all the involved motions of the head of the radius, and you see the arm is fully extended. If passive motion is kept up every day there will be no recurrence of the stiffening of the elbow joint. If passive motion should be omitted for a week, then an anæsthetic will have to be administered again, and we would have to go through the same process. There is a constant tendency to recurrence in cases of contractured tendons if passive motion is not kept up. I think that if alternate flexion and extension of the forearm are maintained twice a day for a period of thirty days there will be no stiffness in the arm.

FIBRO-SARCOMA OF THE CAPSULE OF THE LIVER.

This man is sixty years of age, and has had the swelling which you see since the third of January. He says it came on suddenly. On inspection of the abdomen of this patient we see a tumor distinctly projecting above the surface. We will try to find out its nature. It may be—indeed it is probable—that this tumor was imperceptible to the patient in its early stages, and that he first noticed it about the first of January. We will now percuss the abdomen for the purpose of distinctly locating the tumor. You remember that where there is resonance we have the intestines with the gaseous contents, and where there is flatness there is a solid viscus or tumor. This swelling is directly over the region of the liver. It feels hard and firm. At first it seems almost as if it were cartilaginous in its character, and I find difficulty in separating it from the cartilage above.

Grasping the edges of the ribs in the way you see, I can now make a distinct separation between the tumor and the ribs; it passes under the ribs and is on the side of the liver. It is not movable, it is adherent. Notice the man's color. You see he is not jaundiced; the tumor there does not involve the tissues of the liver proper, because we would have some evidence of liver disease. The conjunctivæ are clear and the skin is white, not jaundiced in any degree, so that we can exclude the parenchyma of the liver from being the seat of the tumor. It is doubtless a tumor springing from the capsule of the liver—probably a fibro-sarcoma. The absence of pain is frequently found in the fibrous form of sarcomatous tumors. It depends very much upon the situation in which they are found. I am satisfied from an examination of this tumor, that we have to deal here with a fibro-sarcoma of the capsule of the liver. You will remember that sarcoma always springs from connective tissue; that carcinoma proper comes from epithelial structures. The kind of structure called the mesoblastic is that from which sarcomata spring. We may expect the tumor to enlarge steadily until it will encroach upon the internal vicera to such an extent that his digestion will be interfered with, and it will then pursue a very rapid course. I advise the patient to have an exploratory operation but he declines.

